# Comprehensive analysis of repetitive extragenic palindrome sequences identified in bacteria and archaea using a new web-based tool, RepRanger

**DOI:** 10.1128/msphere.00124-25

**Published:** 2025-07-07

**Authors:** Oleg N. Murashko, Connor Morgan-Lang, Chen-Hsin Albert Yu, Hsin-Nan Lin, Anna Chao Kaberdina, Shin-Yu Kung, Vladimir R. Kaberdin, Sue Lin-Chao

**Affiliations:** 1 Institute of Molecular Biology, Academia Sinica 71562, Taipei, Taiwan; 2 Koonkie Cloud Services Inc., Menlo Park, California, USA; 3 Department of Immunology, Microbiology and Parasitology, University of the Basque Country UPV/EHU, Leioa, Spain; 4 Basque Foundation for Science, IKERBASQUE 197447, Bilbao, Spain; 5 Research Centre for Experimental Marine Biology and Biotechnology (PIE-UPV/EHU) 684055 , Plentzia, Spain; The University of Iowa, Iowa City, Iowa, USA

**Keywords:** palindromic element, extragenic repetitive sequences, consensus motif, small noncoding RNA, post-transcriptional regulation

## Abstract

Repetitive extragenic palindromic (REP) sequences were first discovered in the intergenic regions of *Escherichia coli* and later found in phylogenetically distinct bacterial species. However, their biological roles and sequence conservation remain unclear. Considering the growing number of microbial genome sequencing and annotation projects, along with the lack of online tools for REP identification, we developed a new web-based platform, RepRanger, to rapidly identify and annotate putative palindromic elements, including REPs. Using RepRanger, we identified >4,000 REPs in the *E. coli* MG1655 genome. Moreover, 81 (~52%) of its 157 small noncoding RNAs (sRNAs; known to regulate gene expression) contain REPs. Further analysis using RepRanger, alongside other bioinformatics tools, yielded 10 REP consensus motifs. Notably, REPs potentially involved in translational control (i.e., located within 15 nucleotides downstream of an open reading frame) primarily host motif 4, whereas REPs present in sRNAs predominantly encoded motif 9. Putative targets of REP-containing sRNAs include genes that rewire metabolic pathways in response to environmental changes, indicating that REPs likely contribute to bacterial adaptation. We also reveal that REP consensus motifs are similar across pathogenic and environmental *E. coli* strains but not commensal and laboratory ones. We employed RepRanger to search for REPs and confirm that they are present across bacteria and archaea. Furthermore, we uncovered similarities in REP sequences between bacterial and archaeal genomes. Thus, we demonstrate that RepRanger represents a versatile tool for discovering REPs in bacteria and archaea, providing new insights into their functions and sequence diversity.

**IMPORTANCE:**

Repetitive extragenic palindromic (REP) sequences were first discovered in *Escherichia coli*, but their biological roles, diversity, and sequence conservation remain unclear. We have developed a web-based tool, RepRanger, to identify and annotate putative palindromic elements, including REPs. Using RepRanger, we identified approximately 4,000 REPs in the *E. coli* MG1655 genome. We show that >50% of small noncoding RNAs (sRNAs) contain REPs. The predicted functions of REP-containing sRNAs indicate that REPs likely contribute to bacterial environmental adaptability. In addition, we have discovered REPs in pathogenic, environmental, and commensal *E. coli,* allowing us to assess their sequence similarity. We show that REPs are widely present in bacterial and archaeal genomes and share some sequence similarities. Our comparison of REPs in annotated genomes broadens the current understanding of REP sequence diversity, conservation, and function.

## INTRODUCTION

Palindromic elements (PEs) contain inverted sequences that can fold into stable stem-loop secondary structures that are present in the genomes of all domains of life as well as in viruses. In bacteria, PEs include Rho-independent terminators (RITs) (1), repetitive extragenic palindromic elements (REPs) (for a review, see reference 2), and clustered regularly interspaced short palindromic repeat (CRISPR) sequences (for a review, see reference 3), all of which are mainly located in intergenic regions and are transcribed together with flanking genes. REPs were first found ~40 years ago in the enterobacteria *Escherichia coli* and *Salmonella typhimurium* (4), and then were subsequently identified and characterized in many other bacterial species (for a review, see reference 5). RNA sequencing revealed that nearly 80% of the 355 *E. coli* REPs annotated in the EcoCyc database (https://ecocyc.org) by 2015 are transcribed under aerobic conditions (6). REPs can be defined as a class of repetitive sequences that are (i) usually transcribed from extragenic regions (i.e., they seldom overlap with protein-coding regions) (4), (ii) palindromic (i.e., they form stem-loop structures) (4), (iii) relatively short (e.g., 14–40 nucleotides [nt] in *E. coli*; according to the EcoCyc database), (iv) highly similar (i.e., possess a common consensus motif) (7, 8), and (v) present in multiple copies per genome, depending on the species (for a review, see reference 5). *E. coli* REPs annotated in the EcoCyc database can be classified into two groups based on their location in an operon, i.e., 3*'*-terminal (in mono- and polycistronic operons) or intergenic (flanked by two coding regions of a polycistronic operon).

The preferential location of REP elements in close proximity to the gene ends has given rise to some hypotheses regarding their physiological roles, origin, and evolution (2). REPs in DNA are involved in chromosome organization, DNA topology, and nucleoid structures (9–11), with nucleoid DNA condensation potentially being mediated by REP-containing RNA. In particular, it has been reported that components of the REP325 element and at least one of its RNA products (nucleoid-associated noncoding RNA 4) play a role in bacterial nucleoid DNA condensation (6). Furthermore, several studies have indicated that the hairpin structure of a REP element in RNA can serve as a transcriptional attenuator, acting as a barrier to ribonucleolytic degradation and, hence, stabilizing REP-associated mRNAs and their intermediates (12–15). For instance, both *in vitro* and *in vivo* analyses have revealed that deleting REPs from the 3*'*-terminal untranslated regions of *E. coli* mRNAs reduces their half-life (15). Recently, ATP depletion was shown to stabilize REP-containing transcripts, based on whole-genome transcriptomics of the effect of fluoride treatment on *E. coli* gene expression under anaerobic conditions (16).

It has also been reported previously that nearly half of all *E. coli* REP sequences have the potential to stall ribosomes immediately upstream of the termination codon, resulting in endonucleolytic cleavage of the mRNA (17). In addition to the association of REPs with mono- and polycistronic mRNAs, they have also been identified within bacterial regulatory RNAs, such as small noncoding RNAs (sRNAs) (18). These sRNAs typically repress translation of target mRNAs by pairing with the ribosome-binding site, competing with initiating ribosomes, and often triggering mRNA decay (19). sRNAs are highly structured RNA molecules of 50–500 nucleotides, including *trans*-encoded sRNAs typically encoded within intergenic regions (20). Currently, 88 sRNAs are annotated in the EcoCyc database. Three *E. coli* sRNAs (SroC, C0362, and C0664) containing REP elements have already been discovered (21, 22). SroC destabilizes the sRNA GcvB and may act as a “sponge” by interacting with it (23). The functions of the other two REP-containing sRNAs, C0362 and C0664, remain unknown.

Given the important function of REPs in regulating genes, along with the ever-increasing number of bacterial genome sequencing and annotation projects and the availability of public bioinformatics tools, we felt that a web-based REP search platform would significantly facilitate our understanding of REP-related functions. The previously published ExtraTrain tool (24) is no longer accessible online. Consequently, we developed a new web-based tool, RepRanger (https://bc.imb.sinica.edu.tw/RepRanger/index.html), which can rapidly identify and annotate putative PEs, including REPs, in all publicly available annotated bacterial and archaeal genomes. Through comprehensive RepRanger-assisted analysis, we have discovered REPs in different phyla of bacteria and archaea, defined their consensus motifs, and described their occurrence and similarity across species. Overall, RepRanger can be utilized to enhance our understanding of REP sequence diversity, conservation, and potential functions across various genomes.

## RESULTS

### Development and use of the web-based tool RepRanger to predict and annotate candidate REPs

**Fig 1:**
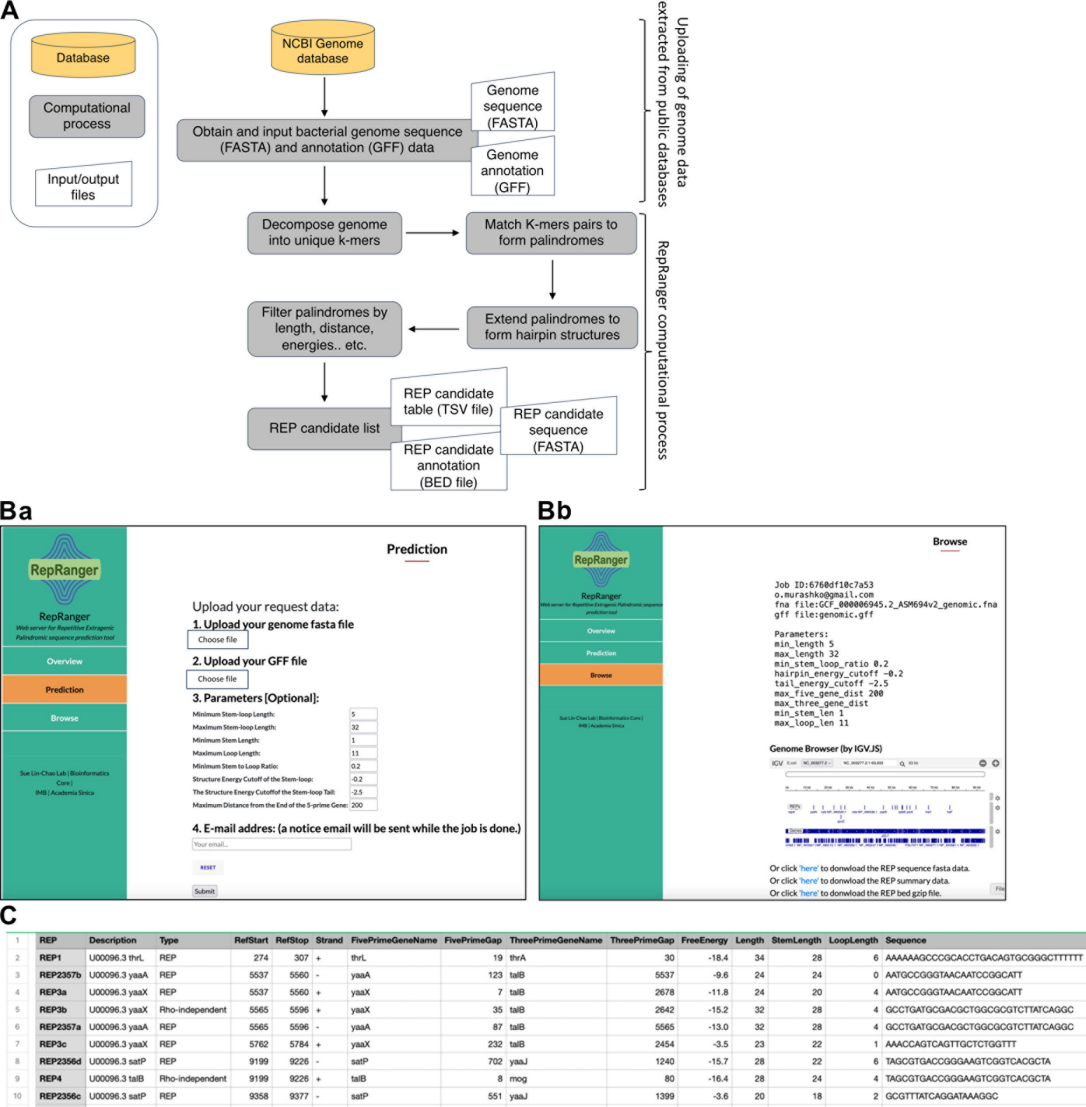
Workflow and use of the web-based tool RepRanger: (**A**) workflow for identifying candidate REPs in a genome, (**B**) screenshots showcasing the RepRanger user interface, including the prediction (Ba) and browse (Bb) pages, and (**C**) example of an output table (TSV file) generated by RepRanger.

Although REP elements were discovered a long time ago, their biological roles, occurrence across many bacterial taxa, and sequence conservation all remained poorly defined. To fill these knowledge gaps, we have developed a novel web-based tool named RepRanger (Repetitive Extragenic Palindrome Ranger), which is aimed at facilitating prediction of REPs in the intergenic regions of annotated bacterial and archaeal genome sequence data. The RepRanger webserver (https://bc.imb.sinica.edu.tw/RepRanger/index.html) was developed in PHP and Python scripts. The workflow processes bacterial or archaeal genome sequence (FASTA) and annotation (GFF) data files from the NCBI Genome Database (https://www.ncbi.nlm.nih.gov/datasets/genome/) to generate output files containing candidate REPs according to the workflow depicted in Fig. 1A. A number of parameters (e.g., stem and/or loop length, stem-to-loop ratio, energy cutoff of the stem-loop or tail, and distance from the 5*'* end of the gene) can be adjusted via the RepRanger web interface (Fig. 1Ba), where users can also input an email address to which a link to the results will be sent. RepRanger employs a multistep computational approach to identify palindromic sequences within the intergenic regions of a genome (see Materials and Methods for details), including decomposing genomes into k-mers, matching k-mer pairs to form palindromes, inferring hairpin structures, etc. These palindromic sequences are filtered based on the thermodynamics of RNA folding heuristics, the presence of uridine-rich tails characteristic of RITs, and several structural criteria pertaining to REPs (see Materials and Methods for details), ultimately generating a list of candidate REPs. This list can be visualized via the embedded Genome Browser (IGV.JS [25]) or downloaded in TSV, BED, and FASTA formats (Fig. 1Bb). The TSV file (Fig. 1C) contains detailed annotation information of candidate REPs, including position, length, distance from the upstream and downstream open reading frames (ORFs), free energy, and sequence, among other data.

### Genome-wide prediction and analysis of REP candidates in *Escherichia coli* MG1655 using RepRanger

**Fig 2:**
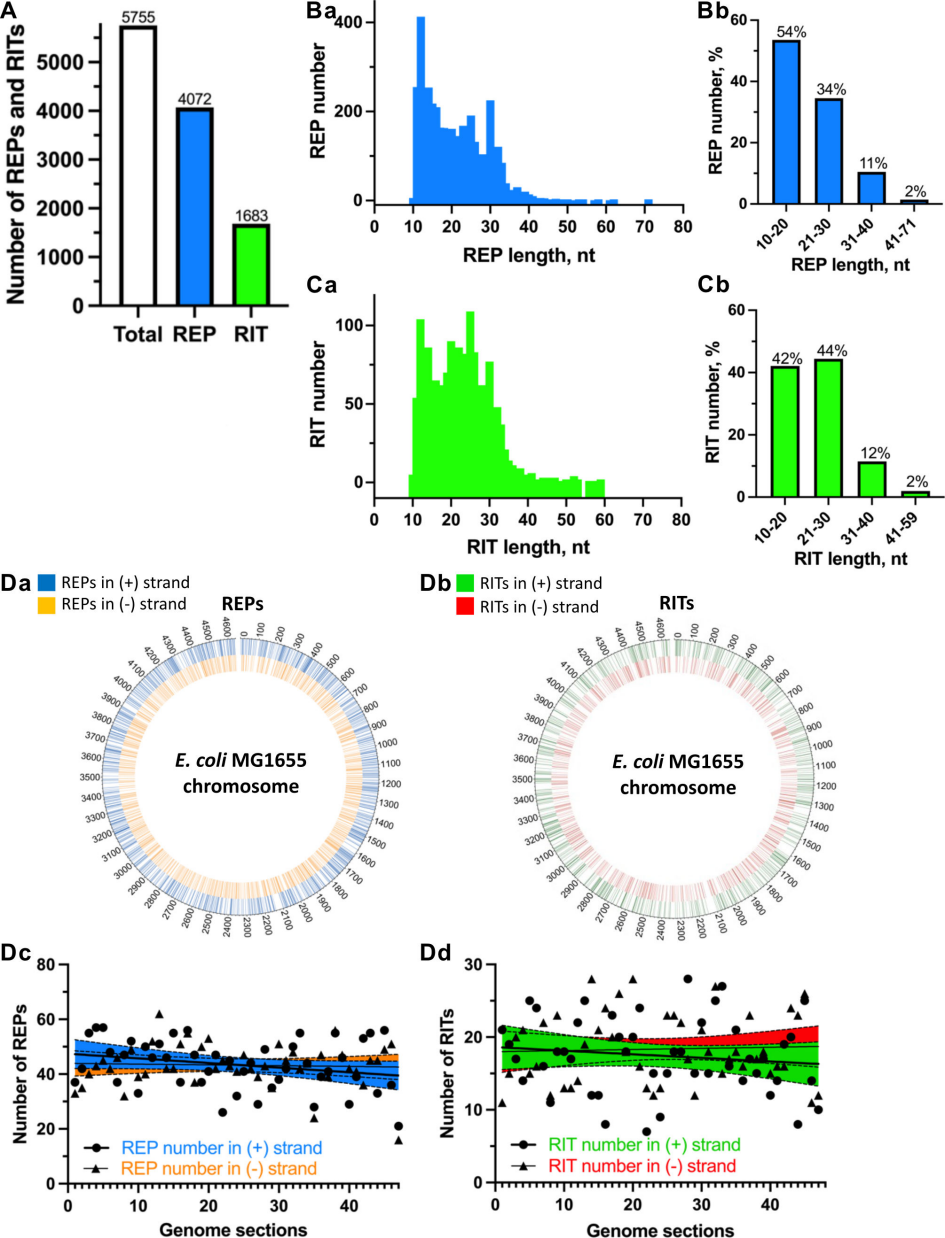
Putative REPs and RITs in *E. coli* MG1655, as identified by RepRanger, as well as their length distributions and chromosomal locations. Numbers (A) of REPs and RITs in the *E. coli* MG1655 genome, as well as their length distributions (Ba, b and Ca, b). (Da and b) Diagrams of the localizations of REPs and RITs in the *E. coli* genome, and (Dc and d) their number distribution per 100 kb of DNA on the (+) and (−) strands. The number of REPs or RITs is indicated for each section of the genome (47 in total; each represents a 100 kb region, with the 47th section representing 41,652 bp).

To test our newly developed tool, RepRanger, we used it to predict PE elements in the best-annotated bacterial genome, *E. coli* K-12 substrain MG1655 (26). According to the latest *E. coli* genome annotation (NCBI Reference Sequence: NC_000913.3), the maximum length of spacer/intergenic regions (i.e., regions potentially containing REPs) separating collinear genes is 6,176 base pairs (bp). Consequently, in RepRanger parameters, we used a maximum distance of 6,200 nucleotides between a REP and the upstream gene to ensure that our analysis encompassed all intergenic regions. We set 10 nt as the minimum cutoff length for PEs and other parameters (see Materials and Methods for details). RepRanger-assisted prediction identified 5,755 REP candidates, including 4,072 REPs (~71%) and 1,683 RITs (~29%) (Fig. 2A).

Further analysis of the predicted elements revealed that the lengths of REPs and RITs ranged from 10 to 71 nt (Fig. 2Ba) and 10 to 60 nt (Fig. 2Ca), respectively. Despite some differences in length distribution patterns, the vast majority of the predicted REPs and RITs (~88% and ~86%, respectively) ranged from 10 to 30 nt in length (Fig. 2Bb and Cb, respectively), which is consistent with previously reported ranges (7, 8). Next, we explored if REPs and/or RITs are distributed uniformly across the genome or show signs of clustering or periodicity. We found that REPs and RITs were distributed throughout the entire genome on both the (+) and (−) strands, with no clear evidence of clustering or periodicity (Fig. 2Da and b). Specifically, the number of REPs (per 100 kb of DNA) varied from 26 to 57 (95% confidence interval [CI]: ~42 to ~53) on the (+) strand and from 24 to 62 (95% CI: ~39 to ~49) on the (−) strand (Fig. 2Dc). In comparison, the number of RITs varied from 7 to 28 (95% CI: ~16 to ~22) on the (+) strand and from 11 to 28 (95% CI: ~15 to ~21) on the (−) strand (Fig. 2Dd). However, there are regions on the chromosome that contain a number of REPs or RITs falling well outside the 95% CI (Fig. 2Dc and d).

### Identity and occurrence of REP consensus motifs in *Escherichia coli* REPs

**Fig 3:**
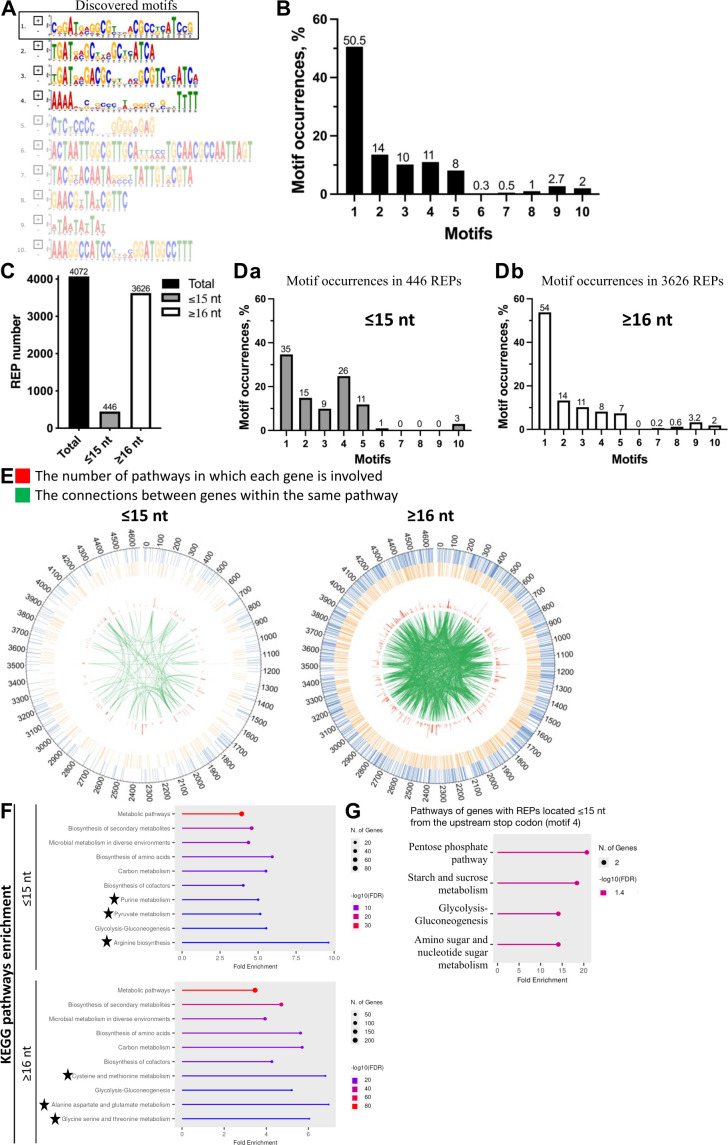
REP consensus motifs identified in *E. coli* MG1655 generated by the MEME tool, the genomic location of REPs potentially involved in translational control, and gene ontology (GO) analysis of REP-containing genes. (**A**) REP consensus motifs discovered in the *E. coli* MG1655 genome based on MEME analysis. Motifs with an *E*-value >0.05 are shown as partially transparent. Motif 1, which is similar to previously proposed motifs (7, 8), is highlighted by a black rectangle. (**B**) Occurrences of the REP consensus motifs. We used the FIMO web tool to calculate occurrences of the motifs (*P*-value <0.05). (**C**) Numbers of REPs located ≤15 or ≥16 nt from an upstream stop codon and (Da and b) the occurrences of the REP consensus motifs. (**E**) Locations of REPs along the *E. coli* chromosome. The red histograms in the plot represent the numbers of pathways that each gene is involved in, with green lines showing the links between genes that are in the same pathway. (**F**) Functional enrichment analysis of genes with REPs located ≤15 and ≥16 nt from an upstream stop codon. (**G**) Pathways of genes with REPs located ≤15 nt from the upstream stop codon (motif 4). Functional enrichment analysis was performed using the ShinyGO tool (http://bioinformatics.sdstate.edu/go/) with a false discovery rate (FDR) cutoff of 0.05. Fold Enrichment: measures the magnitude of enrichment (higher values indicate stronger enrichment and are an important metric of effect size). Pathway Genes: the total number of genes in a pathway or GO term. Pathways specifically associated with genes containing REPs located ≤15 or ≥16 nt from an upstream stop codon are indicated by black stars.

The first consensus motif for *E. coli* REPs has been proposed as GC(g/t)GATGGCG(g/a)GC(g/t) … (g/a)CG(c/t)CTTATC(c/a)GGCCTAC, based on approximately 30 annotated REPs (8). Subsequently, analysis was extended to all 356 REP elements annotated in EcoCyc, which revealed the following consensus sequence GCCGGATGCGGCGTGAACGCCTTATCCGGCCTACGA (7) that overlaps with the first reported motif (8). Advances in annotating the *E. coli* genome have led to a significant increase in the number of new REPs. Here, RepRanger identified a total of 4,072 REPs, which is approximately sevenfold more than the 697 REPs annotated in EcoCyc, indicating that all of these REPs do not necessarily have the same consensus motif. To test this idea, we employed the MEME web tool (27) and uncovered 10 consensus motifs (Fig. 3A) among the 4,072 REPs identified by RepRanger, with the top four hits having an *E*-value of less than 0.05. Our motif 1 is similar to the motif reported previously (7, 8), but we describe nine novel REP motifs for the first time. Next, we analyzed the occurrence of all 10 of these motifs among the 4,072 REPs using the FIMO web tool (28), which revealed that motif 1 displayed the highest occurrence (~50.5%) among the analyzed motifs (Fig. 3B). The variety of REP consensus motifs implies that they may be involved in different biological functions beyond currently known translational control (17) (see results below).

### Identification of REPs potentially involved in translational control in *E. coli*

A previous study revealed that a significant proportion of REPs is located 1–20 nt away from the 3′ end of the nearest ORF and demonstrated that REPs can downregulate translation but only if they are positioned fewer than 16 nt from the stop codon (17). To search for REPs predicted by RepRanger that may function in translational control, we first categorized the REPs into two groups: (i) those with a distance from stop codon not exceeding 15 nt (i.e., putative translational regulators) and (ii) those located ≥16 nt away (unknown functions). We found that only 446 out of 4,072 putative REPs (~11% of the *E. coli* REPs identified) belonged to the first group (Fig. 3C) and, therefore, may regulate the translation of upstream genes, while the remaining 3,626 (~89%), i.e., the majority of REPs, belonged to the second group and may have other functions.

Since we identified a much higher number of REPs with a distance from the stop codon not exceeding 15 nt (*n* = 446) than previously found (17), first we examined if REPs that potentially downregulate translation possess a unique consensus motif. As shown in Fig. 3Da and b, REPs hosting motifs 1 (~35%) and 4 (~26%) proved the most common among the putative translation-linked REPs (*n* = 446; Fig. 3Da), with motif 1 also displaying the highest occurrence among the remaining 3,626 REPs (~54%; Fig. 3Db). Subsequently, we analyzed the intergenic locations of REPs on the *E. coli* chromosome and determined the biological pathways in which each adjacent gene is involved (by EcoCyc) as well as the specific connections between genes in the same pathway (Fig. 3E).

In order to understand the function of the genes associated with the REPs in these two groups, we employed the ShinyGO web tool (http://bioinformatics.sdstate.edu/go/) (Fig. 3F). The REPs located within 15 nt of the nearest ORFs (group 1) were specifically associated with purine/pyruvate metabolism and arginine biosynthesis (Fig. 3F). The REPs located ≥16 nt away from their nearest ORF (group 2) were specifically associated with the metabolism of cysteine, methionine, alanine, aspartate, glutamate, glycine, serine, and threonine (Fig. 3F).

In addition, our data show that motif 4 (Fig. 3Da and b) is the most common within putative translation-linked REPs, and it occurred in ~3.3-fold more frequently in group 1 than in group 2. The ShinyGO web tool revealed that the vast majority of genes with motif 4 in group 1 control carbohydrate metabolism (Fig. 3G).

### Over half of sRNAs contain REPs

**Fig 4:**
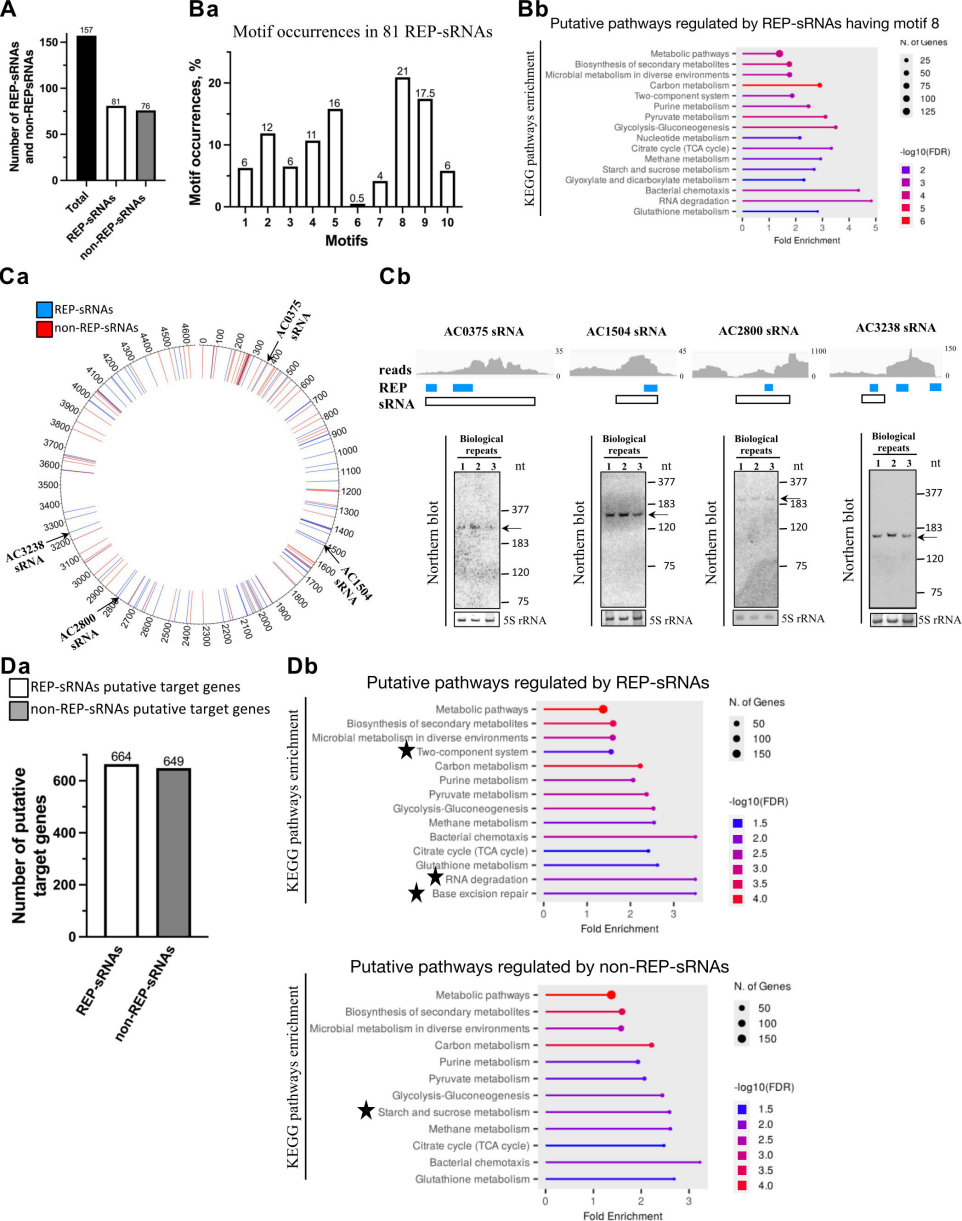
REPs associated with *E. coli* MG1655 sRNAs, their consensus motifs, and putative biological pathways regulated by these sRNAs. (**A**) Number of REP-hosting and non-REP-harboring sRNAs and (Ba) occurrences of 10 REP consensus motifs (see Fig. 3A) in the REP-hosting sRNAs. (Bb) Putative pathways regulated by REP-hosting sRNAs sharing the most abundant motif 8. Location (Ca) of REP-hosting sRNAs and non-REP-harboring sRNAs along the *E. coli* chromosome. The sRNAs for which expression has been validated are shown (black arrow). (Cb) Validation of predicted REP-hosting sRNA expression via Northern blot analysis. A comparison of reads corresponding to mapped small RNAs in the *E. coli* genome is shown at the top of each Northern blot panel. The *y*-axis represents the number of RNA-seq reads. REPs are shown in blue, and sRNAs are represented by an empty rectangle, respectively. Hybridizations were performed with probes specific for the selected sRNAs under aerobic growth conditions. The 5S rRNA served as an internal loading control. The expected sizes (in nucleotides) of the full-length sRNAs are indicated. The molecular ladder was obtained by hybridizing total RNA with radiolabeled probes specific for *rnpB* (M1) RNA (377 nt), 6S RNA (183 nt), 5S rRNA (120 nt), and tRNA-Asn (75 nt). Three biological replicates were performed, and representative images are shown. (Da) Number of REP-hosting sRNA and non-REP-harboring sRNA putative target genes, and (Db) putative pathways regulated by REP-hosting and non-REP-harboring sRNAs. Pathways specifically regulated by REP-hosting or non-REP-harboring sRNAs are indicated by black stars.

REP sequences can also be found within some regulatory RNAs, such as sRNAs that play an important role in bacterial stress adaptation (18). To date, 88 sRNAs have been annotated in the EcoCyc database, and 3 of them (SroC, C0362, and C0664) have been identified as containing REP elements. Given the regulatory roles of sRNAs, we postulate that REPs may have broader biological significance. Since the sRNAs subjected to our analysis do not contain an ORF and therefore were not annotated in the RepRanger output files, they were mapped to the *E. coli* genome using the Integrative Genomics Viewer (IGV) program (https://igv.org/doc/desktop/). This analysis uncovered that 51 (~58%) of the sRNAs host REPs (Table 1). In fact, the number of sRNAs that contain REPs might be considerably higher. Our recent analysis (29) of RNA sequencing profiles obtained for total RNA extracted from wild-type *E. coli* MG1655 made it possible to identify new putative sRNAs expressed in this strain (see Materials and Methods, and Table S1). Among the 69 newly identified sRNAs in the current study, we found that 30 (~44%) contain REPs. Collectively (Fig. 4A), we have found REPs in almost 52% (*n* = 81) of *E. coli* sRNAs (i.e., from among the 88 EcoCyc-annotated sRNAs and 69 newly identified ones).

**TABLE 1:** Association of predicted REPs and RITs with sRNAs

Number	sRNA	REP/RIT
1	sokC	–^*a*^
2	sroA	–
3	sgrS	REP
4	ftsO	–
5	tff	–
6	ispZ	–
7	eyeA	–
8	nc1	REP
9	sraA	REP
10	chiX	REP
11	ipeX	REP
12	sokE	–
13	sroC	REP
14	chiZ	–
15	sdhx	–
16	zbiJ	REP
17	rybA	RIT
18	rybB	REP
19	sraB	REP
20	C0293	REP
21	narS	–
22	rttR	REP
23	mcaS	REP
24	fnrS	REP
25	ralA	–
26	micC	REP
27	rydC	REP
28	SokB	REP
29	mgrR	–
30	dicF	–
31	rydB	RIT
32	rprA	REP
33	ryeA	REP
34	sdsR	REP
35	micL	REP
36	3'ETS leuZ	REP
37	sdsN	REP
38	dsrA	REP
39	rseX	–
40	isrC	REP
41	sibA	REP
42	sibB	REP
43	cyaR	REP
44	orzP	REP
45	micF	–
46	ryeG	REP
47	sroE	REP
48	timR	RIT
49	ryfA	REP
50	glmY	REP
51	ohsC	REP
52	ryfD	–
53	raiZ	REP
54	micA	REP
55	sokX	–
56	csrB	RIT
57	gcvB	REP
58	omrA	–
59	omrB	–
60	sibC	REP
61	och5	–
62	sibD	–
63	sibE	REP
64	sraG	REP
65	arcZ	REP
66	ryhB	–
67	agrA	REP
68	agrB	–
69	arrS	REP
70	gadF	REP
71	gadY	–
72	rirA	–
73	istR	RIT
74	rbsZ	RIT
75	gImZ	REP
76	esrE	–
77	spf	RIT
78	csrC	–
79	cpxQ	REP
80	oxyS	REP
81	sroH	REP
82	malH	REP
83	pspH	REP
84	ryjA	–
85	aspX	REP
86	GO-10706	–
87	ryjB	REP

^*a*^ – indicates that REP/RIT was not found in the sRNA sequence.

We anticipate that some sRNAs are descended from the same original REP element, and therefore, certain REP motifs could be relevant to sRNA regulation. We analyzed the occurrences of all 10 motifs (see Fig. 3A) in the 81 REP-hosting sRNAs (Fig. 4Ba) and found that motifs 5, 8, and 9 were the most common in the sRNA-associated REPs, with motif 8 displaying the highest occurrence (~21%). In addition, we assessed the sRNAs whose REP contains motif 8 (i.e., that with the highest occurrence) and observed that their targets control many genes involved in central carbon metabolism, biosynthesis of secondary metabolites, microbial metabolism in diverse environments, among others (Fig. 4Bb).

We selected four of the newly discovered REP-hosting sRNAs to validate their genomic location (Fig. 4Ca), and their expression was confirmed by Northern blotting (Fig. 4Cb).

An increased number of REP-containing sRNAs may also enhance the number of putative targets controlled by these sRNAs. Indeed, a comparison of the sRNA targets identified by TargetRNA software (30) revealed 664 target genes for REP-hosting sRNAs and 649 target genes for non-REP-harboring sRNAs (Fig. 4Da). Further analysis using the ShinyGO web tool revealed that REP-hosting sRNA target genes are specifically associated with controlling two-component systems, RNA degradation, and base excision repair, whereas the non-REP-harboring sRNA target genes apparently regulate starch and sucrose metabolism (Fig. 4Db).

### REP consensus motifs are similar across pathogenic and environmental *E. coli* strains but not in commensal or laboratory strains

With the aim of gaining insight into REP occurrence in the genus *Escherichia* of Family Enterobacteriaceae, we analyzed their presence and sequence similarity across the genomes of all major groups of *E. coli* pathogens (Table 2) (31) as well as each group of nonpathogenic *E. coli* strains (i.e., commensal, environmental, and laboratory). More specifically, we used RepRanger to analyze three representative annotated genomes from each of those groups (Table 2).

**TABLE 2:** Pathogenic and nonpathogenic *E. coli* strains used in this study, their genome size and genome assembly, GC content, and number of REPs and RITs

Category	Type	Organism	Genome size, Mb	Gene number	GC, %	REP number	RIT number	Genome assembly
Pathogenic	Pathotype							
Intestinal	Enteropathogenic (EPEC)	*Escherichia coli* EPEC 2827	5.5	5,706	50.5	4,224	1,766	ASM174272v1
		*Escherichia coli* EPEC 2081	5.2	5,399	50.5	4,094	1,710	ASM174270v1
		*Escherichia coli* EPEC 393	5.3	5,325	50.5	3,926	1,736	ASM128199v1
	Shiga toxin producing (STEC)	*Escherichia coli* STEC 2868	5.3	5,346	50.5	4,133	1,818	ASM128172v1
		*Escherichia coli S*TEC 487	5.7	5,993	50.5	4,333	1,946	ASM160976v1
		*Escherichia coli* STEC 931	5.7	5,950	50.5	4,374	1,957	ASM160707v1
	Verocytotoxin producing	*Escherichia coli* BCW5746	5.0	5,126	50.5	3,928	1,685	BCW5746
		*Escherichia coli O157:H-*	5.7	5,736	50.5	4,621	2,034	ASM1686443v1
		*Escherichia coli* ED180	5.2	5,425	50.5	3,872	1,753	ED180_contigs
	Enterohemorrhagic	*Escherichia coli O157:H7*	5.9	5,986	50.5	4,734	2,113	ASM169551v1
		*Escherichia coli O145:H28 str.* RM13514	5.7	5,750	50.5	4,811	2,048	ASM52003v1
		*Escherichia coli O111:H8 strain* 110512	5.6	5,667	50.5	4,762	2,109	ASM799065v1
	Enterotoxigenic (ETEC)	*Escherichia coli* E925	5.2	5,197	50.5	4,620	1,910	L1_E925
		*Escherichia coli* E1373	5.2	5,078	50.0	4,439	1,877	L7_E1373_ETEC
		*Escherichia coli* ETEC H10407	5.3	5,321	50.5	4,535	1,878	ASM21047v1
	Enteroinvasive	*Escherichia coli* EF432	5.0	4,876	50.5	4,082	1,759	PDT000946469.1
		*Escherichia coli* O124	5.0	4,976	50.5	3,676	1,616	PDT000948272.1
		*Escherichia coli* V73	5.2	5,053	50.5	4,105	1,786	PDT001024289.1
	Enteroaggregative	*Escherichia coli* 1091	5.1	4,961	50.5	3,776	1,637	PDT000981904.1
		*Escherichia coli* 146052	5.3	5,293	50.5	3,877	1,717	PDT000131993.2
		*Escherichia coli* 153145	5.1	5,106	50.5	3,869	2,123	PDT000132361.2
	Diffuse adhering (DAEC)	*Escherichia coli* DAEC F-1845	5.3	5,226	50.5	4,110	1,763	ASM2254447v1
		*Escherichia coli* MEX-1	5.1	5,461	51.0	4,173	1,764	ASM1336841v1
		*Escherichia coli* MEX-7	5.0	5,213	51.0	4,113	1,749	ASM1336004v1
	Adherent invasive (AIEC)	*Escherichia coli* HM605	5.3	5,238	50.5	4,281	1,882	ASM28537v1
		*Escherichia coli* AIEC17	5.0	5,148	50.5	3,861	1,709	AIEC17
		*Escherichia coli* LF82	4.8	4,627	50.5	3,964	1,691	ASM28449v1
Extraintestinal	Uropathogenic (UPEC)	*Escherichia coli* CFT073	5.2	5,097	50.5	4,348	1,879	ASM1426294v1
		*Escherichia coli* UTI89	5.2	5,056	50.5	4,206	1,822	ASM1326v1
		*Escherichia coli* UPEC-245U/190328	5.3	5,247	50.5	4,450	1,938	UTI-2_GENOME
	Neonatal meningitis	*Escherichia coli O18 str.* F10018-41	4.7	4,564	51.0	3,521	1,480	PDT001681196.1
		*Escherichia coli* RS218	5.8	5,142	50.5	4,078	1,790	ASM81734v1
		*Escherichia coli* IHE3034	5.1	5,024	50.5	4,090	1,806	ASM2574v1
	Avian pathogenic (APEC)	*Escherichia coli* APEC O2	4.9	4,875	50.5	3,495	1,457	ASM162037v1
		*Escherichia coli* APEC 16-1068	5.3	5,250	50.5	4,245	1,881	ASM2875267v1
		*Escherichia coli* APEC O18	5.0	4,887	50.5	4,027	1,766	APECO18
	Sepsis associated	*Escherichia coli* Z1002	5.6	5,558	50.5	4,662	2,054	ASM214267v1
		*Escherichia coli* Z247	5.4	5,310	50.5	4,410	1,906	ASM214271v1
		*Escherichia coli* ST1193	5.1	4,980	50.5	3,984	1,718	PDT001778707.1
Nonpathogenic	Serotype							
	Commensal	*Escherichia coli* Nissle 1917	5.1	4,878	50.5	4,218	1,798	ASM2155983v1
		*Escherichia coli HS*	4.6	4,422	51.0	3,935	1,709	ASM2245360v1
		*Escherichia coli* SE15	4.8	4,672	50.5	4,021	1,775	ASM1048v1
	Environmental	*Escherichia coli* SMS-3-5	5.2	5,037	50.5	4,431	1,856	ASM1964v1
		*Escherichia coli* ED1	5.2	5,023	50.5	4,033	1,774	ASM1265896v1
		*Escherichia coli O104:H4 str.* C227-11	5.4	5,400	50.5	4,536	2,005	ASM98676v1
	Laboratory	*Escherichia coli* MG1655	4.6	4,639	51.0	4,072	1,683	ASM584v2
		*Escherichia coli* BL21(DE3)	4.6	4,453	51.0	3,998	1,661	ASM1316701v1
		*Escherichia coli* DH5alpha	4.5	4,437	50.5	3,691	1,582	ASM98243v1

**Fig 5:**
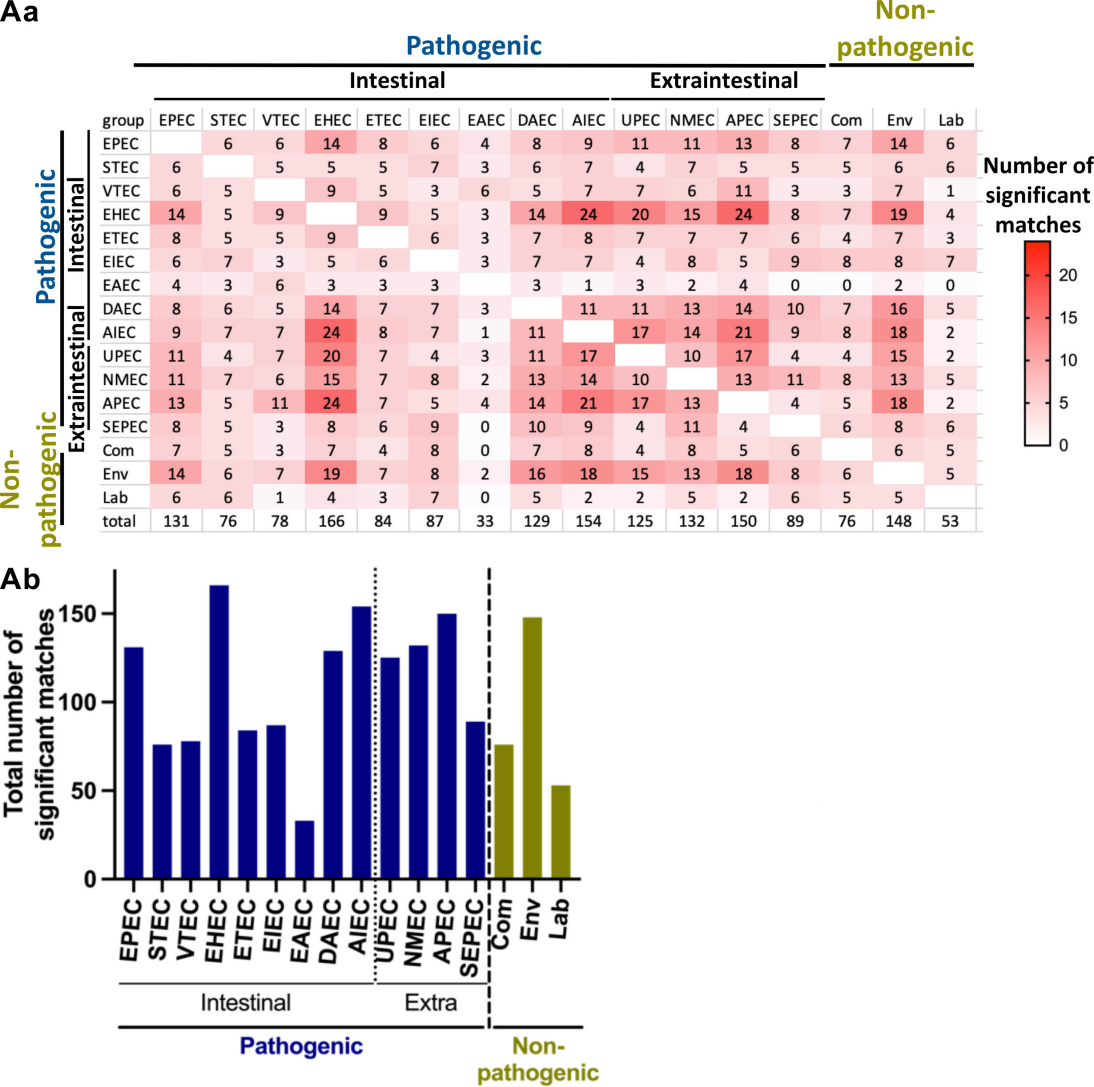
Motif similarity in pathogenic and nonpathogenic *E. coli* strains. (Aa) Motif similarity among pathogenic and nonpathogenic *E. coli* strains and (Ab) total number of significant matches of the motifs. We used the TomTom web tool to calculate the similarity of the motifs (*P*-value <0.001) identified using MEME. Pathogenic *E. coli:* EPEC, enteropathogenic; STEC, Shiga toxin producing; VTEC, verocytotoxin producing; ETEC, enterotoxigenic; EIEC, enteroinvasive; DAEC, diffuse adhering; AIEC, adherent invasive; UPEC, uropathogenic; NMEC, neonatal meningitis associated; SEPEC, sepsis associated.

Since *E. coli* strains can be found in diverse environments, it is possible that REPs present in bacteria originating from the same environment share common motifs. The REP consensus motifs we identified in enterohemorrhagic *E. coli* (EHEC; intestinal pathogenic strains), avian pathogenic *E. coli* (APEC; extraintestinal pathogenic strains), and environmental nonpathogenic strains are highly conserved in other groups of *E. coli* (Fig. 5Aa and b). Enteroaggregative (EAEC) strains presented the highest number of REPs with significant similarity to those identified in the other *E. coli* strains we analyzed. Together, these data indicate that REP consensus motifs are similar across pathogenic and environmental *E. coli* strains but differ from those found in commensal and laboratory strains.

### REPs and RITs are widely distributed across bacteria and archaea

**Fig 6:**
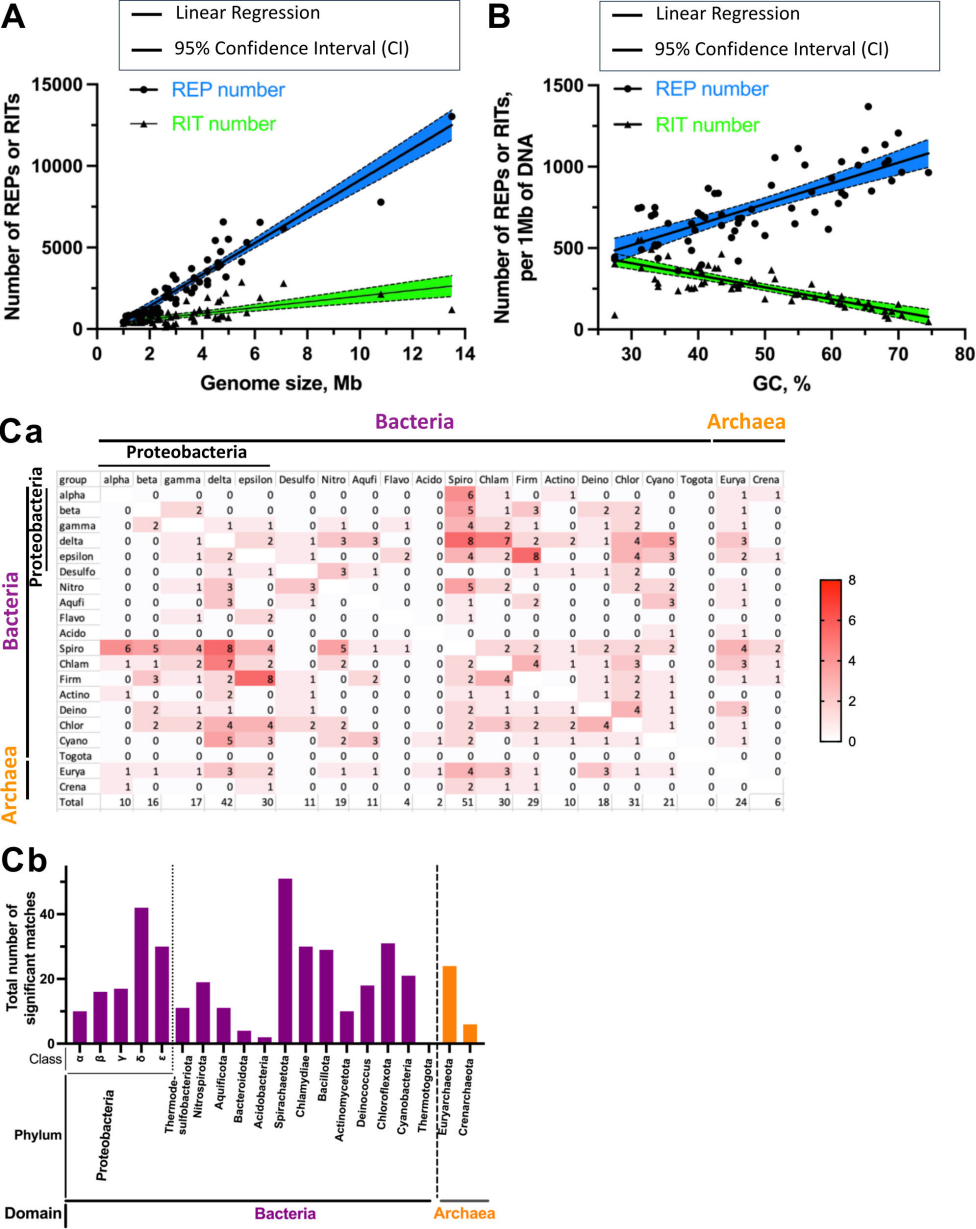
Numbers of putative REPs and RITs discovered by RepRanger in different bacteria and archaea and REP motif similarity. (**A**) REPs and RITs normalized to the size or GC content (**B**) of the cognate genomes. Each black dot or triangle represents one species of bacteria or archaea, respectively. The confidence bands above and below the fitted line represent 95% confidence intervals (in blue and green for REPs and RITs, respectively). (Ca) Motif similarity among the bacterial and archaeal strains assessed in this study and (Cb) total number of significant matches of the motifs. We used the TomTom web tool to calculate the similarity of the motifs (*P*-value <0.001) identified using MEME.

In order to compare the number of REPs between members of bacteria and archaea, we used RepRanger to identify putative REPs in representative genomes from the two domains. Some phyla have a small number of species and available annotated NCBI sequencing data; therefore, only major phyla with sufficient annotated NCBI sequencing data were used. Three species per phylum were selected for analysis, as defined in a previous study (32), as well as from each class within the Pseudomonadota (formerly Proteobacteria). We found that REPs and RITs are widely distributed across bacteria and archaea, and their numbers of REPs and RITs were largely proportional to the size of the corresponding genome (Fig. 6A).

Bacteria are known for the variability of GC content in their genomes. Since GC pairs are generally more stable than AT pairs, it has been proposed that GC-rich genomes are better adapted to high temperatures than AT-rich ones (33). Consequently, the increased stability and functionality of PEs with higher GC content may also promote their retention in organisms having GC-rich genomes. We compared the numbers of REPs and RITs in bacteria and archaea having high or low GC content. As illustrated in Fig. 6B, the number of REPs is proportionally higher in GC-rich genomes, whereas the number of RITs appears to decrease with increasing genomic GC content. Examples including *Baaleninema simplex* (Cyanobacteria), *Pyrolobus fumarii* (Crenarchaeota), and *Lichenicola cladoniae* (Alphaproteobacteria) had 1,055 (51.5% GC), 1,112 (55.0% GC), and 1,369 REPs (65.5% GC), respectively, while *Borrelia bissettiae* (Spirochetota) had 89 RITs (27.5% GC).

### Comparison of REP consensus motifs reveals relatively low sequence similarity across bacterial and archaeal genomes

Our analysis of REPs indicates that certain motifs could potentially be different in phylogenetically distant organisms, i.e., bacteria and archaea. To evaluate sequence conservation of the REPs identified in bacteria and archaea using RepRanger, we again employed MEME to identify the consensus motifs in the REP sequences, which were then compared using TomTom (34) (Fig. 6Ca and b). We found that REPs identified in Spirochaetota display the greatest motif similarity to those found in other bacterial and archaeal species (Fig. 6Ca and b). In contrast, motifs shared among REP sequences in the Thermotogota exhibit no similarity to those of other bacteria and archaea. The REP motifs found in delta-Proteobacteria presented the highest total number of significant matches to REP motifs of other Proteobacteria, whereas those of alpha-Proteobacteria exhibited the lowest similarity. In terms of archaea, Euryarchaeota exhibited higher motif similarity to other taxa than Crenarchaeota (Fig. 6Ca and b).

## DISCUSSION

PEs are encoded in the genomes of organisms belonging to all domains of life. Although bacterial genomes are smaller and more compact than those of eukaryotes, they still contain a substantial number of PEs, which perform various biological functions (35, 36). Among them, REP elements are small palindromic sequences of mostly 20–50 nt in length. Numerous REPs have already been found in bacterial genomes, ranging from ~700 in *E. coli* MG1655 (697 REPs, https://ecocyc.org) to thousands in some *Pseudomonas* strains (37). REPs have been implicated in a variety of cellular functions, including genome structuring and plasticity, modulation of stress responses as well as the regulation of gene expression at both transcriptional and post-transcriptional levels (2, 8, 10, 12, 17, 38–40). However, previous experimental data and bioinformatics analyses have not yet provided a complete understanding of the biological roles of REPs. Exploring the biological functions of REPs is complicated by the fact that they have only been annotated in a few model organisms, and their presence in different classes of bacteria and archaea has not been examined systematically.

Given the growing number of whole-genome sequences available for a diverse range of distantly related microorganisms, it is now possible to identify and compare various regulatory elements across different bacterial and archaeal species. Previously, REPs had been identified in >80 species of 8 different prokaryotic phyla (41). Here, we used our novel RepRanger tool to search for REP-like sequences in the genomes of 16 different bacterial and archaeal taxa, corresponding to the main branches of the latest phylogenetic trees (32). Our analysis has revealed that REPs and RITs are widespread in both those domains of life (Table 3).

**TABLE 3:** Bacterial and archaeal strains used in this study, their genome size and genome assembly, GC content, and number of REPs and RITs

Kingdom and phylum	Class	Organism	Genome size, Mb	Gene number	GC, %	REP number	RIT number	Genome assembly
Bacteria								
Pseudomonadota (formerly Proteobacteria)	Alpha	*Lichenicola cladoniae* JCM 33604	4.8	5,724	65.5	6,569	782	ASM1320107v1
		*Hyphomonas jannaschiana* VP2	3.6	3,554	61.5	3,707	737	SOAPdenovo v1.05
		*Erythrobacter cryptus* DSM 12079	3.0	2,899	68.0	3,055	348	ASM42298v1
	Beta	*Iodobacter ciconiae*	3.9	3,549	48.0	2,879	1,201	ASM395234v1
		*Alcaligenes faecalis*	4.2	3,871	57.0	3,552	1,170	ASM96730v2
		*Tepidimonas ignava*	2.7	2,644	69.0	2,466	301	ASM434262v1
	Gamma	*Psychrobacter arcticus*	2.7	2,265	43.0	2,260	1,017	ASM1230v1
		*Escherichia coli* MG1655	4.6	4,639	51.0	4,072	1,683	ASM584v2
		*Inmirania thermothiophila*	2.6	2,546	74.5	2,506	125	ASM375163v1
	Delta	*Desulfotalea psychrophila*	3.7	3,259	46.5	2,529	1,040	ASM2594v1
		*Pyxidicoccus fallax*	13.5	10,871	70.5	13,027	1,199	ASM1293365v1
		*Hippea alviniae* EP5-r	1.7	1,824	37.0	612	404	ASM42038v1
	Epsilon	*Sulfurimonas gotlandica* GD1	3.0	2,921	33.5	1,557	931	ASM24291v2
		*Helicobacter pylori*	1.6	1,557	39.5	975	411	ASM1782153v1
		*Caminibacter pacificus*	1.9	2,041	34.0	987	546	ASM508398v2
Thermodesulfobacteriota		*Thermodesulfatator atlanticus*	2.3	2,279	45.0	1,297	577	ASM42158v1
		*Thermosulfurimonas dismutans*	2.1	2,171	50.0	1,211	395	ASM165258v1
		*Caldimicrobium thiodismutans*	1.8	1,832	38.5	978	468	ASM154827v1
Nitrospirota		*Nitrospira moscoviensis*	4.6	4,576	62.0	3,788	635	ASM127377v1
		*Leptospirillum ferriphilum*	2.6	2,603	54.0	1,940	459	LFTS_HGAP3_1
		*Thermodesulfovibrio yellowstonii* DSM 11347	2.0	2,060	34.0	873	527	ASM2098v1
Aquificota		*Hydrogenothermus marinus*	1.6	1,728	27.5	710	647	ASM368866v1
		*Aquifex aeolicus* VF5	1.6	1,820	43.5	1,022	468	ASM862v1
		*Hydrogenivirga caldilitoris*	1.8	1,947	46.0	1,175	494	ASM366400v1
Bacteroidota		*Flavobacterium urumqiense*	3.4	3,100	33.5	2,411	1,745	IMG-taxon 2617270781
		*Flavobacterium ginsenosidimutans*	5.5	4,745	33.5	4,118	2,884	ASM3747810v1
		*Thermoflavifilum aggregans*	2.8	2,410	46.0	1,903	728	ASM279773v1
Acidobacteriota		*Granulicella aggregans*	5.7	4,666	60.0	5,307	1,009	ASM2568556v1
		*Acanthopleuribacter pedis*	10.8	6,706	57.5	7,776	2,149	ASM1737785v1
		*Chloracidobacterium thermophilum B*	3.7	3,103	61.5	3,109	576	ASM22629v1
Spirochaetota		*Leptospira stimsonii* Yale	4.7	4,342	42.5	3,926	1,775	ASM354587v1
		*Borrelia bissettiae*	1.0	970	27.5	432	356	Borrelia crocidurae str. 03-02
		*Spirochaeta thermophila* DSM 6578	2.6	2,371	61.0	2,012	412	ASM18434v2
Chlamydiota		*Chlamydia abortus*	1.1	1,017	40.0	789	418	G1_hybrid
		*Chlamydia pneumoniae*	1.2	1,093	40.5	842	430	ASM720v1
		*Chlamydia psittaci*	1.2	1,036	39.0	782	431	ASM20425v1
Bacillota		*Bacillus subtilis*	4.2	4,536	43.5	2,955	1,948	ASM904v1
		*Acetivibrio clariflavus*	4.9	4,228	35.5	3,197	2,220	ASM23708v1
		*Fervidicola ferrireducens*	2.4	2,593	46.0	1,007	610	ASM156242v1
Actinomycetota		*Arthrobacter livingstonensis*	5.0	4,633	65.0	5,512	705	ASM321981v1
		*Acidipropionibacterium thoenii*	2.9	2,699	68.0	3,293	246	ASM42344v1
		*Acidimicrobium ferrooxidans*	2.2	2,107	68.5	2,283	152	ASM2326v1
Deinococcota		*Deinococcus alpinitundrae*	4.7	4,854	64.0	4,744	810	ASM998289v1
		*Deinococcus aerius* TR0125	4.5	4,446	70.0	5,433	704	ASM289737v1
		*Calidithermus roseus*	3.7	3,754	66.0	3,149	482	ASM357409v1
Chloroflexota		*Dehalococcoides mccartyi*	1.4	1,464	48.5	912	405	ASM83090v1
		*Anaerolinea thermolimosa*	4.2	3,751	54.5	2,724	904	ASM105019v2
		*Ardenticatena maritima*	3.6	3,083	59.5	2,214	789	ASM130617v1
Cyanobacteria		*Trichormus variabilis* 0441	7.1	5,974	41.5	6,156	2,805	ASM985660v1
		*Baaleninema simplex*	6.2	5,373	51.5	6,543	1,936	ASM33235v1
		*Rubidibacter lacunae* KORDI 51-2	4.2	3,524	56.0	4,240	857	KS51_v1
Thermotogota		*Mesotoga prima*	3.0	2,700	45.5	1,814	824	ASM14771v3
		*Defluviitoga tunisiensis*	2.1	1,905	31.5	1,030	793	DTL3
		*Fervidobacterium changbaicum*	2.3	2,116	40.5	914	648	ASM411707v1
Archaea								
Euryarchaeota		*Methanocaldococcus bathoardescens*	1.6	1,714	31.0	1,190	876	ASM73906v1
		*Pyrococcus furiosus* DSM 3638	1.9	2,123	41.0	1,307	554	ASM824508v1
		*Methanocaldococcus vulcanius* M7	1.8	1,785	31.5	1,349	991	ASM2462v1
Crenarchaeota		*Pyrolobus fumarii*	1.8	2,012	55.0	2,002	395	ASM22339v1
		*Candidatus Culexarchaeum yellowstonense*	2.1	2,529	39.0	1,020	526	ASM2470702v1
		*Candidatus Methanomethylicia archaeon*	1.6	1,879	33.0	1,117	788	ASM3887967v1

We used RepRanger to search for palindromic elements in the genome of the model organism *E. coli* MG1655. We found approximately sixfold and approximately fivefold more REPs and RITs (Fig. 2A), respectively, in the *E. coli* MG1655 genome compared to those annotated in the EcoCyc database. The close proximity of some REPs to a stop codon may indicate that they are involved in regulating translation, especially given the fact that REPs occurring within 15 nt of an upstream stop codon stall ribosomes (17). This latter study proposed that 260 of the 496 REP sequences identified by the authors act in translational regulation. In the current study, RepRanger identified 446 of 4,072 REPs as meeting the criterion of being within 15 nt of a stop codon and thus likely to contribute to regulating translation (Fig. 3C), including the previously reported *nrdAB* REP-containing transcript (17). Nevertheless, although we identified a larger number of putative translation-regulating REPs, the proportion of all *E. coli* REPs we identified is ~1.7 times smaller than that reported previously (17), likely due to the larger REP data set we generated herein. Further experimental work will be necessary to test if all of these REPs are indeed involved in translational control.

Some REPs might play essential roles in regulating gene expression at the post-transcriptional level. Previous studies have shown that stable structures formed by REPs can serve as a barrier to RNA degradation by exoribonucleases, thereby stabilizing mRNA transcripts and their decay intermediates (13, 15). Our RepRanger tool could prove useful in detecting upstream and downstream fragments stabilized by REPs and/or RITs and in identifying the distribution and frequency of potential RNase cleavage sites in their vicinity.

One criterion for identifying REPs is their strong sequence similarity, which is characterized by a shared consensus motif. However, the REP sequence consensus motif(s) is not yet well defined. Although an initial consensus sequence (based on ~30 *E*. *coli* REPs) of GC(g/t)GATGGCG(g/a)GC(g/t) … (g/a)CG(c/t)CTTATC(c/a)GGCCTAC was proposed almost 40 years ago (8), a previous positional analysis using a considerably larger number (356) of annotated REP elements revealed that 224 of them share a slightly different GCCGGATGCGGCGTGAACGCCTTATCCGGCCTACGA motif (7). There are currently 697 REPs annotated in the EcoCyc database. It is likely that as the number of identified REPs increases, the number of conserved motifs and their consensus sequences will change. In the present study, we identified even more REPs and determined consensus motifs across the entire set of *E. coli* REPs. We used the MEME web tool (27) to assess REP sequence similarity and found that we could cluster the 4,072 putative *E. coli* MG1655 REPs into 10 groups based on distinct motifs, with the top 4 hits having an *E*-value of ≤0.05 (see Fig. 3A). Among these 10 motifs, motif 1, which is shared by ~50.5% of the 4,072 REPs (Fig. 3B), is very similar to previously proposed motifs (7, 8), whereas the other motifs appear to be novel.

In addition to being present in coding transcripts, we also identified REPs within sRNAs, both those annotated in the EcoCyc database and newly identified sRNAs in this study (see Table S1), all of which may exert essential roles in bacterial adaptation to stress. Using RepRanger, we found that in addition to the three known sRNAs hosting REP sequences already annotated in the EcoCyc database (i.e., SroC, C0362, and C0664), several additional sRNAs host REPs. In fact, our analysis uncovered that 81 sRNAs (~52%) in *E. coli* MG1655 are associated with REPs belonging to 10 different groups and that the REP sequences in each of those groups possess similarity to a specific motif. Moreover, REP sequences showing similarity to motifs 5, 8, and 9 proved the most common in our data set, indicating that these consensus sequences might help sRNAs to exert their functions. Nevertheless, the functions of REPs in sRNAs require further study.

We also assessed the conservation of REP sequences across several pathogenic and nonpathogenic *E. coli* strains. Although REPs are present in all of the pathogenic strains we analyzed (see Table 2), the REPs in a few strains (i.e., EHEC, APEC, and environmental strains) appear to share sequence similarity with many REP sequences of other pathogenic strains (Fig. 5Aa and b). We observed that the REP consensus motifs we have identified are similar among pathogenic and environmental *E. coli* strains but differ from those found in commensal and laboratory strains.

Furthermore, using RepRanger combined with other bioinformatics tools enabled us to predict and classify bacterial and archaeal REP motifs based on their similarity (Fig. 6Ca and b). Interestingly, we uncovered high sequence similarity of Spirochaetota REPs to those identified in other bacterial taxa. In contrast, Thermatogota REPs showed no sequence similarity to those of other bacteria or archaea. Prior to the current study, published information on the REPs in those strains had been lacking.

In summary, our findings demonstrate the high versatility of RepRanger in facilitating the discovery and analysis of PEs in the genomes of two domains of life (bacteria and archaea). We found that ~52% of the stress-related sRNAs in *E. coli* MG1655 possess REPs. Moreover, our extensive analysis of the PEs present in bacteria and archaea has revealed that REPs are widespread in both kingdoms of life and allowed us to uncover consensus motifs shared by different groups of these genetic elements.

## MATERIALS AND METHODS

### Prediction of putative REP candidates by RepRanger

To find candidate REPs in a genome’s intergenic regions, the following steps are taken. First, the intergenic regions are broken into k-mer sequences (default *k* = 6). Next, palindromic k-mer pairs are identified and filtered based on a distance threshold (50 bp). These sequences are then extended in both directions until a mismatch is encountered, and then adjacent k-mer pairs are assembled if the mismatch gaps are below a certain length (default = 2). Finally, REPs are distinguished from Rho-independent transcription terminators by the presence of thymine-rich tails. Specifically, RITs are characterized by distinct structural features—short stem-loop hairpin formations immediately followed by a thymine-rich sequence at their 3′ end. In this study, RITs were identified using the methodology described in equation 1 of the TransTermHP method, as detailed by Kingsford et al. (42). This equation calculates a “tail score,” which assigns greater weight to thymine residues located within the 15 nucleotides at the 3′ tail of the hairpin structure (weighting parameters: *T* = 0.9; others = 0.6). The threshold for the tail energy score is set at −2.5. In addition to this tail score threshold, all other parameters used to evaluate the energy of potential hairpin structures were directly adopted from the TransTermHP method. These parameters, detailed in Table 1 of Kingsford et al. (42), include specific energy values for nucleotide pairings (such as G-C, A-T, and G-T), as well as penalties for mismatches, gaps, and loop formations [Loop_pen(n) = 1 × (*n* − 2)]. The specific energy values used were G-C pairing at −2.3, A-T pairing at −0.9, G-T pairing at 1.3, a mismatch penalty of 3.5, and a gap penalty of 6.0. All thresholds utilized in our methodology for predicting RITs were adopted directly from the Kingsford et al. study. Their comprehensive approach was originally applied to predict terminators across 343 bacterial and archaeal genomes, representing the full collection of complete prokaryotic genomes available in GenBank at the time. Our study adheres to these established and validated parameters to enable robust terminator identification. Therefore, we adopted the above well-established criteria (including threshold values) to filter out RITs and classified all remaining PE elements as putative REPs.

The default parameters used in RepRanger—such as palindrome length (minimum: 5 nt, maximum: 32 nt), loop length (maximum: 11 nt), stem length (minimum: 1 nt), distance to the gene 5′ end (maximum: 200 nt), stem-to-loop length ratio (minimum: 0.2), mismatch gap between adjacent k-mer pairs (maximum: 2 bp), energy cutoff for hairpin structures (–0.2 kcal/mol), and tail energy cutoff (–2.5 kcal/mol)—are derived from the 697 annotated REPs in the EcoCyc database and ensure that the identified palindromes are both relevant and structurally suitable to function as REPs.

For our analysis, we selected a minimum cutoff length of 10 nt for PEs, based on the length required to form a minimal secondary structure (Fig. S1), as predicted using the RNAfold web server (http://rna.tbi.univie.ac.at/cgi-bin/RNAWebSuite/RNAfold.cgi).

### Prediction and comparison of REP consensus motifs

We classified REP sequences into two categories: (i) pathogenic and nonpathogenic *E. coli*, and (ii) other bacteria and archaea. We then used the MEME Suite (27) to generate motifs for the identified REP sequences. MEME employs the expectation-maximization algorithm to discover novel ungapped motifs in unaligned sequences. In the expectation step, it calculates the probability of each possible motif occurrence in the sequences, given the current motif model, and it updates the motif model to maximize the likelihood of the observed sequences in the maximization step. The expectation and maximization steps are repeated iteratively until convergence.

Next, we used TomTom (34) to compare these motifs between two groups. TomTom ranks the motifs based on their similarity, providing *P*-values (default: 0.001) to indicate the statistical significance of the matches. This comparison helps to identify similar motifs among REP groups, thereby providing insights into their potential biological functions.

We estimated similarity scores between any two REP groups according to three variables:

- Na = number of unique motifs in group A.
- Nb = number of unique motifs in group B.
- Nab = number of similar motifs identified between groups A and B.

The similarity score was then defined as Nab/(Na + Nb).

### Novel sRNA prediction

We established the following criteria for identifying new putative sRNAs using an RNA-seq data set published previously (NCBI GEO accession no. GSE189154) (29):

- sRNA length is ~50–400 nt;
- located in intergenic regions (20);
- possible overlap with the coding region of annotated genes;
- expression profile of each putative sRNA is different from the flanking genes/regions;
- sRNA expression levels are different under differential growth conditions (i.e., aerobic vs microaerobic) or different genetic background (i.e., MG1655 wild type vs Rned823-850 mutant);
- binds to the RNA chaperone Hfq (43).

### Bacterial strain, growth condition, RNA isolation, and Northern blot analysis

To prepare subcultures from fresh overnight cultures, the *E. coli* MG1655 strain was grown overnight at 37°C for 16 h in M9 medium supplemented with 0.4% glucose and trace elements [462.56 µM H_3_BO_3_, 34.71 µM MnCl_2_, 10.80 µM FeCl_3_, 7.72 µM ZnSO_4_, 3.16 µM CuSO_4_, 2.30 µM (NH_4_)_6_Mo_7_O_24_, 1.68 µM Co(NO_3_)_2_]. A 16-h fresh overnight culture was diluted into 750 mL fresh M9 medium (to OD_460_ = 0.04–0.05) in a 1-L fermentation vessel chamber (Winpact Parallel Fermentation System FS-05-220, Saratoga, CA, USA). To create cultivation conditions, air was continuously pumped into the chamber at a rate of 0.4 L/min (liters per minute). The culture was grown at 200 rpm, 37°C, and maintained at pH 7.0 by automatic titration with sterile 1 M KOH. The culture was harvested at OD_460_ = 0.5–0.6 for further analyses. In brief, 42 mL of culture from multiple biological replicates was collected into 50 mL tubes with 7 mL (1/6 vol) of ice-cold stop solution (5% phenol and 95% ethanol [vol/vol]) for RNA isolation (see details below). Bacterial pellets were harvested following centrifugation at 4,000 × *g*, 4°C for 15 min, and stored at −80°C before use.

Total RNA was extracted as described previously (44). In brief, bacterial pellets were resuspended in 4 mL KJ medium (50 mM glucose, 25 mM Tris-HCl pH 8.0, 10 mM EDTA pH 8.0, 100 mM NaCl), lysed by placing into boiling 4 mL buffer (0.2 M NaCl, 20 mM Tris-HCl pH 7.5, 40 mM EDTA, 0.5% SDS), and boiled in a boiling water bath for 45 s before adding 4 mL of acidic phenol (pH = 4.5). The solution was mixed gently by slowly inverting the tube ~20 times. Total RNA was extracted in aqueous phase by centrifugation at 4,000 × *g*, 4°C for 1 h. The RNA was precipitated in 1 vol of isopropanol and 1/10 vol of 3 M sodium acetate (pH 7.8) at −20°C. All RNA samples were maintained in isopropanol at −20°C before use. When RNA isolation was performed on aliquots, the same volume of culture from the same batch of biological replicates was used for Northern blot analysis.

For Northern blot analysis, RNA was separated on a 7 M urea gel with 6% or 8% polyacrylamide (acrylamide/bis-acrylamide 19:1) in 0.5× Tris-borate-EDTA (TBE) buffer and electrophoresed at 120 V until the xylene cyanol dye had reached 3/4 of the length of the gel. The RNA was transferred onto Zeta-Probe Blotting membranes (Bio-Rad, Hercules, CA, USA) at 400 mA (100 min at 4°C) in 0.5× TBE buffer and cross-linked to the membrane using a Stratalinker 2400 UV Crosslinker (Stratagene). The membrane was pre-blotted with ULTRAhyb Ultrasensitive Hybridization Buffer or ULTRAhyb-Oligo hybridization buffer (Invitrogen) for 2 h at 65°C or 42°C, respectively. An antisense 5′-end [γ−32P]-labeled DNA oligo probe was used to detect target sRNAs. Probe sequences are listed in Table S2. T4 polynucleotide kinase (NEB) was used to generate isotope-labeled probes with [γ-^32^P] ATP. Radioactive probes were purified using a MicroSpin G-25 column (GE Healthcare) before being added into the hybridization buffer for RNA detection at 42°C, respectively, for at least 6 h. Wash solutions (2× or 0.5× saline sodium citrate [SSC], 0.1% sodium dodecyl sulfate [SDS]) were applied to remove nonspecific signals. Northern blot signals were captured using super-resolution BAS Storage Phosphor Screening (GE Healthcare) and visualized with a GE Amersham Typhoon system.

### Bioinformatics

The BioCyc Database Collection (https://biocyc.org) was used to identify REPs in bacteria and archaea. Motif occurrences were scanned using FIMO (28). Circos plots were generated using the *circlize* (v0.4.15) (45) R packages, respectively. The freely available tool ShinyGO v0.66 (http://bioinformatics.sdstate.edu/go/) (46) was used for gene ontology enrichment analysis.

### Quantification and statistical analysis

All statistical tests were performed using GraphPad Prism version 9.0.

## SUPPLEMENTAL MATERIAL

Figure S1 msphere.00124-25-s0001.tif Validation of stem structure.

Supplemental Legends msphere.00124-25-s0002.docx Supplemental table and figure legends.

Table S1 msphere.00124-25-s0003.xlsx Putative sRNAs.

Table S2 msphere.00124-25-s0004.xlsx Oligoprobes.

ASM does not own the copyrights to Supplemental Material that may be linked to, or accessed through, an article. The authors have granted ASM a non-exclusive, world-wide license to publish the Supplemental Material files. Please contact the corresponding author directly for reuse.
