# Supplementary figures and images for: Comprehensive analysis of repetitive extragenic palindrome sequences identified in bacteria and archaea using a new web-based tool, RepRanger
Source: mSphere. 2025 Jul 7;10(7):e00124-25. doi: 10.1128/msphere.00124-25 (PMC12306157; doi:10.1128/msphere.00124-25)

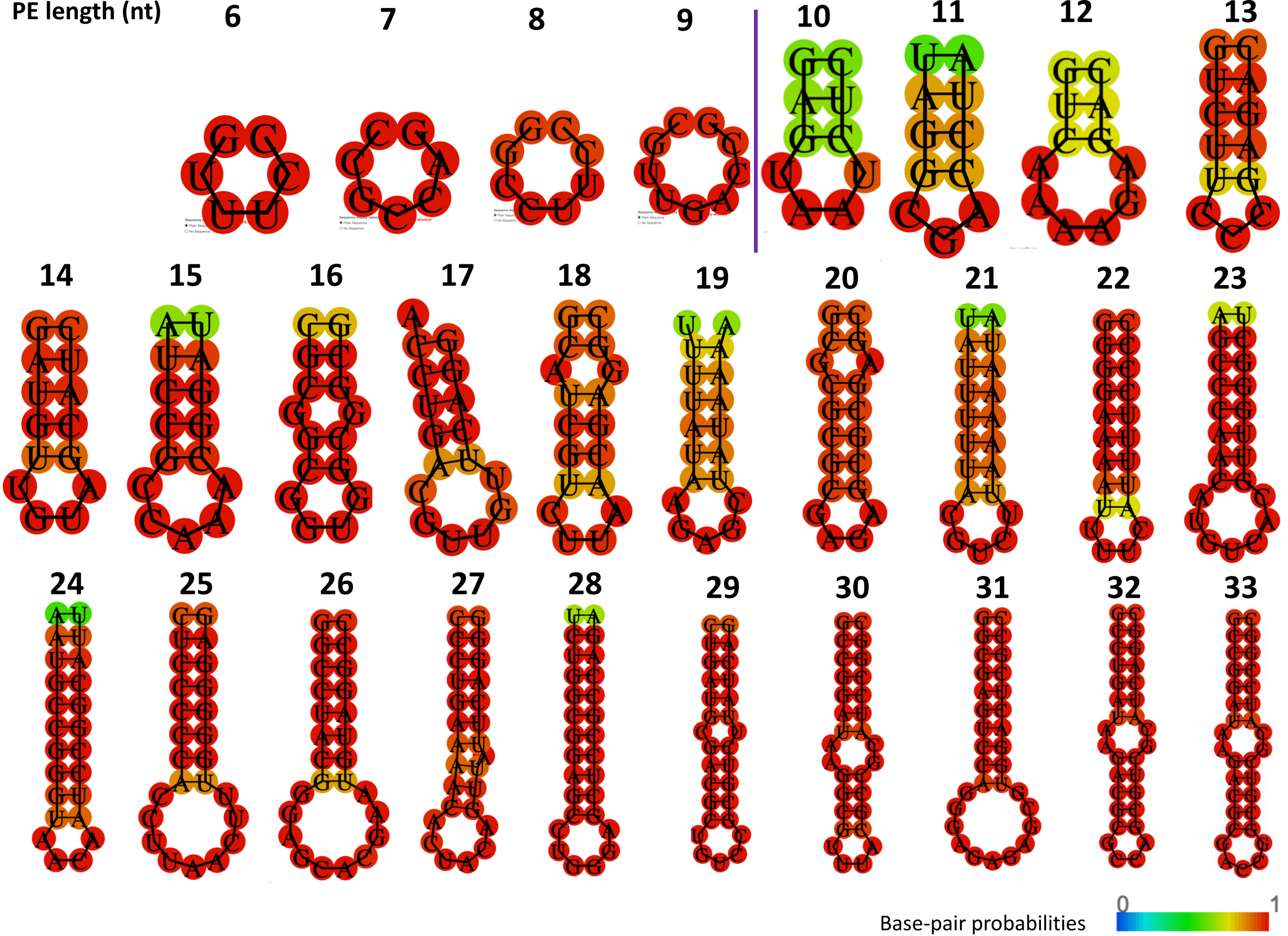

Supplement: Figure S1 — Validation of stem structure. [file msphere.00124-25-s0001.tif]
