# Supplementary material for: Comprehensive analysis of repetitive extragenic palindrome sequences identified in bacteria and archaea using a new web-based tool, RepRanger
Source: mSphere. 2025 Jul 7;10(7):e00124-25. doi: 10.1128/msphere.00124-25 (PMC12306157; doi:10.1128/msphere.00124-25)
Supplement: Supplemental Legends — Supplemental table and figure legends. [file msphere.00124-25-s0002.docx]

**SUPPLEMENTAL FIGURES AND TABLES LEGENDS.**

**Figure S1. Validation of stem structure of PEs (6 to 33 nt in length) identified by RepRanger.** Secondary structure was predicted using the RNAfold web server (<http://rna.tbi.univie.ac.at/cgi-bin/RNAWebSuite/RNAfold.cgi>). Base-pair probabilities from 0 (blue) to 1 (red) are shown.

**Table S1. List of putative sRNAs identified in this study.**

**Table S2. Oligonucleotide primers used for Northern blots.**
